# Evolutionary diversification of cytokinin-specific glucosyltransferases in angiosperms and enigma of missing *cis*-zeatin *O*-glucosyltransferase gene in Brassicaceae

**DOI:** 10.1038/s41598-021-87047-8

**Published:** 2021-04-12

**Authors:** Lenka Záveská Drábková, David Honys, Václav Motyka

**Affiliations:** 1 Laboratory of Pollen Biology, Institute of Experimental Botany of the Czech Academy of Sciences, Rozvojová 263, 165 02 Prague 6, Czech Republic; 2 Laboratory of Hormonal Regulations in Plants, Institute of Experimental Botany of the Czech Academy of Sciences, Rozvojová 263, 165 02 Prague 6, Czech Republic

**Keywords:** Evolution, Molecular evolution

## Abstract

In the complex process of homeostasis of phytohormones cytokinins (CKs), *O*-glucosylation catalyzed by specific *O-*glucosyltransferases represents one of important mechanisms of their reversible inactivation. The CK *O-*glucosyltransferases belong to a highly divergent and polyphyletic multigene superfamily of glycosyltransferases, of which subfamily 1 containing UDP-glycosyltransferases (UGTs) is the largest in the plant kingdom. It contains recently discovered O and P subfamilies present in higher plant species but not in *Arabidopsis thaliana*. The *cis-*zeatin *O*-glucosyltransferase (*cis*ZOG) genes belong to the O subfamily encoding a stereo-specific *O-*glucosylation of *cis-*zeatin-type CKs. We studied different homologous genes, their domains and motifs, and performed a phylogenetic reconstruction to elucidate the plant evolution of the *cis*ZOG gene. We found that the *cis*ZOG homologs do not form a clear separate clade, indicating that diversification of the *cis*ZOG gene took place after the diversification of the main angiosperm families, probably within genera or closely related groups. We confirmed that the gene(s) from group O is(are) not present in *A. thaliana* and is(are) also missing in the family Brassicaceae. However, *cis*ZOG or its metabolites are found among Brassicaceae clade, indicating that remaining genes from other groups (UGT73—group D and UGT85—group G) are able, at least in part, to substitute the function of group O lost during evolution. This study is the first detailed evolutionary evaluation of relationships among different plant ZOGs within angiosperms.

## Introduction

Many aspects of plant growth and development are coordinated by plant hormones. Among them, cytokinins (CKs) constitute one of the major groups, playing a key role in the control of cell division and elongation as well as organogenesis and many other physiological processes in plants. Natural isoprenoid CKs include *N*^6^-(∆^2^-isopentenyl)adenine (iP), *trans*-zeatin (*trans*Z), *cis*-zeatin (*cis*Z) and dihydrozeatin (DHZ) and their derivatives. In the complex process of CK homeostasis, *N*- and *O*-glucosylation represent important mechanisms of CK irreversible and reversible inactivation, respectively, catalyzed by specific *N*- and *O*-glucosyltransferases (for review see e.g. Refs.^1,2^). Products of these conjugation steps, CK *N*- and *O*-glucosides, have been frequently identified in plants^3^, however, their roles in CK biology still remain somewhat unclear.

The CK *O*-glucosyltransferases belong to the large enzyme superfamily of glycosyltransferases (GTs; EC 2.4.x.y)^4^. These enzymes catalyze the transfer of sugar moieties from activated donor molecules to specific acceptor molecules, forming glycosidic bonds. These enzymes can be classified into 92 families, of which family 1 glycosyltransferases, often referred to as UDP glycosyltransferases (UGTs), is the largest in the plant kingdom^4^. A class of UGTs is defined by the presence of a C-terminal consensus sequence and can be found both in the plants and animals^5^. The nomenclature of this polyphyletic multigene family has been recommended based on the evolutionary divergence^6^. In early studies on *Arabidopsis thaliana,* 107 different UGTs were recognized^7^, and 14 distinct groups of UGTs were found^5,8^. However, relationships of some individual UGTs within well-supported subgroups were not strongly resolved due to a very high similarity among closely related sequences and sequenced motifs^4^. Only later research revealed 16 UGT groups based on an analysis of 12 fully sequenced genomes^9,10^.

Biosynthesis of phytohormones CKs in plants starts with transferring an isoprenoid moiety to an adenine present either in nucleotide form (*trans*Z- and iP-type CKs) or bound to *t*RNA (*cis*Z-types). Two distinct origins of the isoprenoid moiety have been reported—(*1*) the methylerythritol phosphate (MEP) pathway localized in plastids and the mevalonate (MVA) pathway in the cytosol^11,12^. The first CK biosynthetic products, iP nucleotides, are further specifically hydroxylated at the *N*^6^-side chain to form *cis*Z or *trans*Z (for review see e.g. Ref.^13^), which may be subsequently conjugated to form corresponding *O*-glucosides. In addition, indirect evidence suggests the existence of a biosynthetic pathway for zeatins without iP intermediates^14^.

Another biosynthetic pathway producing *cis*Z-type CKs involves the release of CKs by a turnover of certain *t*RNAs. Although *cis*Z-type CKs have been reported as essential components of some *t*RNAs in plants^15^, isoprenoid CKs generally occur as structural parts of certain *t*RNA species of all organisms from eubacteria (but not in archaebacteria) to humans^16^. Considering the abundance of *cis*Z-types in the plant kingdom^17^ and early calculations of *t*RNA turnover rates^18^, tRNA degradation does not, however, seem to be a sole pathway for *cis*Z formation in plants.

The *O*-glycosylation of CKs represents a reversible step leading to rapid and efficient CK deactivation. It is catalyzed by *O*-glucosyltransferases that may recognize *trans-*zeatin (*trans*Z), *cis-*zeatin (*cis*Z) and dihydrozeatin (DHZ), i.e. CKs having an available hydroxyl group for glucosylation. The enzymes catalyzing CK *O*-conjugation, *O-*glucosyltransferase (ZOGT, EC 2.4.1.203^19^) and *O*-xylosyltransferase (ZOXT, EC 2.4.1.204^20^) were first reported in the common bean (*Phaseolus vulgaris*) and lima bean (*Phaseolus lunatus*), respectively. Their biochemical characterization showed different CK substrate specificities and sugar donor recognition, and the corresponding genes were cloned by Mok’s group^21,22^. Later on, two genes encoding an *O-*glucosyltransferase specific to *cis*Z (*cisZOG1*, *cisZOG2*) were isolated and characterized in maize^23,24^, and subsequently, three *cis*Z-specific *O-*glucosyltransferases (*cZOGT1*, *cZOGT2*, and *cZOGT3*) were identified from rice^25^. In *Arabidopsis*, CK *O*-glucosylation through *O*-glucosyltransferases is coded by three UGTs (UGT85A1, UGT73C5 and UGT73C1) producing *O*-glucosides with *trans*Z, *cis*Z and DHZ^26^. Although two of these enzymes (UGT73C5 and UGT73C1) exhibit very low activity and utilize also other substrates^27,28^, UGT85A1 represents zeatin *O-*glucosyltransferease with a preference for *trans*Z and substantially contributes to CK homeostasis^29,30^.

Additionally to the findings above, researchers directed their attention to the grass family (Poaceae), namely *Zea mays*, *Sorghum bicolor* and *Oryza sativa*, and examined the evolutionary pattern of gene duplication of the *cisZOG* gene^31^. Having estimated duplication times for *cisZOG* homologs, they found *cisZOG* genes in tandem triplication in rice, five genes in sorghum and one maize gene.

Generally, most genes belong to larger gene families, and the analysis of gene family histories plays an important role in the study of genome evolution. The crucial point is recognition among orthologous genes referring to copies of genes that reveal the phylogeny of species and paralogous genes that evolved by duplication events. For years, knowledge on the organization of the UGTs family was limited to the model plant *Arabidopsis thaliana*. Analysis of this superfamily led to categorization into 54 families, including family 1, which contains UGTs^8^. The largest UGT1 class contains 16 subfamilies. The UDP family expanded during the transition from algae to vascular plants^9^. In this study based on 11 sequenced plant genomes^9^, five phylogenetic groups (A, D, E, G and L) have been recognized to expand more than the others during the evolution of the higher plants (8–50 members depending on plant species). Other groups were represented from 1 to 13 members. In *A. thaliana* group G includes only six members (about 6% of the total UGTs), group H has expanded to become the second most abundant group in this species (18%)^9^. Interestingly, the newly discovered phylogenetic groups O and P^9^ were not found in *Arabidopsis thaliana.* On the other hand, three sequences available in NCBI databases are named *cis*ZOG1-3 and belong to *A. thaliana.*

To address this discrepancy, we directed our attention to group O, which contains unique highly conserved residues in the PSPG motif (Plant Secondary Product Glycosyltransferase; i.e., UGT Prosite consensus^32^) at positions 41 and 42 (His and Ser, respectively) that are not present in any other 1 UGT-glucosyltransferase phylogenetic group. The evolutionary relationships among plant ZOGs are unknown.

Here, we analyzed a large data set of publicly available amino acid sequences with emphasizing the complete genomes of *cis*ZOGs to classify all representatives available across the plant kingdom. We studied different homologous genes belonging to the UGT1 class subfamily O within angiosperms, their domains, motifs, exon/intron organizations and their phylogenetic relationships. In the wide context of angiosperms, comparative analysis of *cis*ZOG phylogeny and protein structural properties allowed us to identify the diversification of two main clades of monocots and eudicots. These main clades could have expanded after divergence from their common ancestor. The wide sampling of *cis*ZOG orthologs and paralogs provides evidence of *cis*ZOG diversification occurring after the diversification of the main angiosperm families, probably within genera or closely related groups. Additionally, we present evidence that the *cis*ZOG gene is not present in *Arabidopsis thaliana* and furthermore is missing in the other members of the family Brassicaceae*.*

## Results

### ZOG gene identification, conserved motif analyses and pairwise similarity approaches endorsing distinct ZOG clades

**Figure 1:**
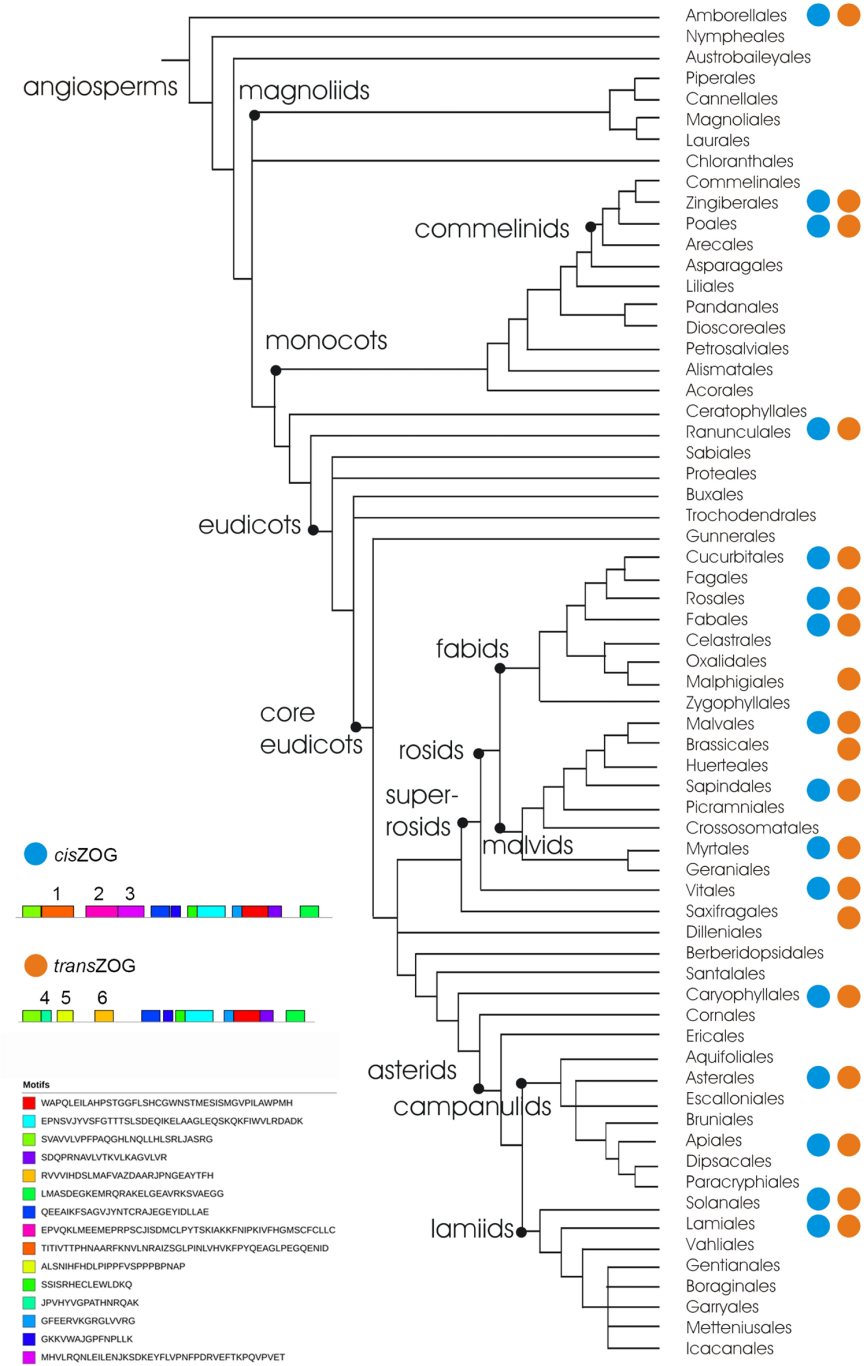
Simplified consensus phylogenetic tree of angiosperms adapted from APG IV. (2016) showing groups where *cis*- or *trans*ZOG were found (based on Phytozome v12) and typical sequence motifs for them. *Cis*ZOG contains conserved motifs 1,2 and 3, *trans*ZOG 4, 5 and 6.

Our combined approach, BLASTP search of fully sequenced genomes via Phytozome v12 and all publicly available *cis-*zeatin *O*-glucosyltransferase homologs from databases, gave us significant results. Homology searches of taxa through databases were crucial for the accuracy of phylogenetic inference and analyses of motifs and domains. From these searches, we did not obtain sequences of Brassicaceae. Compilation of data from completely sequenced genomes allowed us to build a matrix to identify major events of gene duplication and losses in angiosperms during evolution, find *trans-*zeatin O-glucosyltransferase (*trans*ZOG) proteins, and explore Brassicaceae ZOGs and indicate complex outline to *cis*- and *tran*ZOG distribution within angiosperms (Fig. 1). *Cis*ZOG belonging to the phylogenetic group O, which contains the PSPG motif with His and Ser at positions 41 and 42, have typical conserved motifs 1, 2 and 3 (Fig. 1). *Trans*ZOG belonging to the group D contains nine same motifs as *cis*ZOG, but typically have motifs 4, 5 and 6 (Fig. 1).

**Figure 2:**
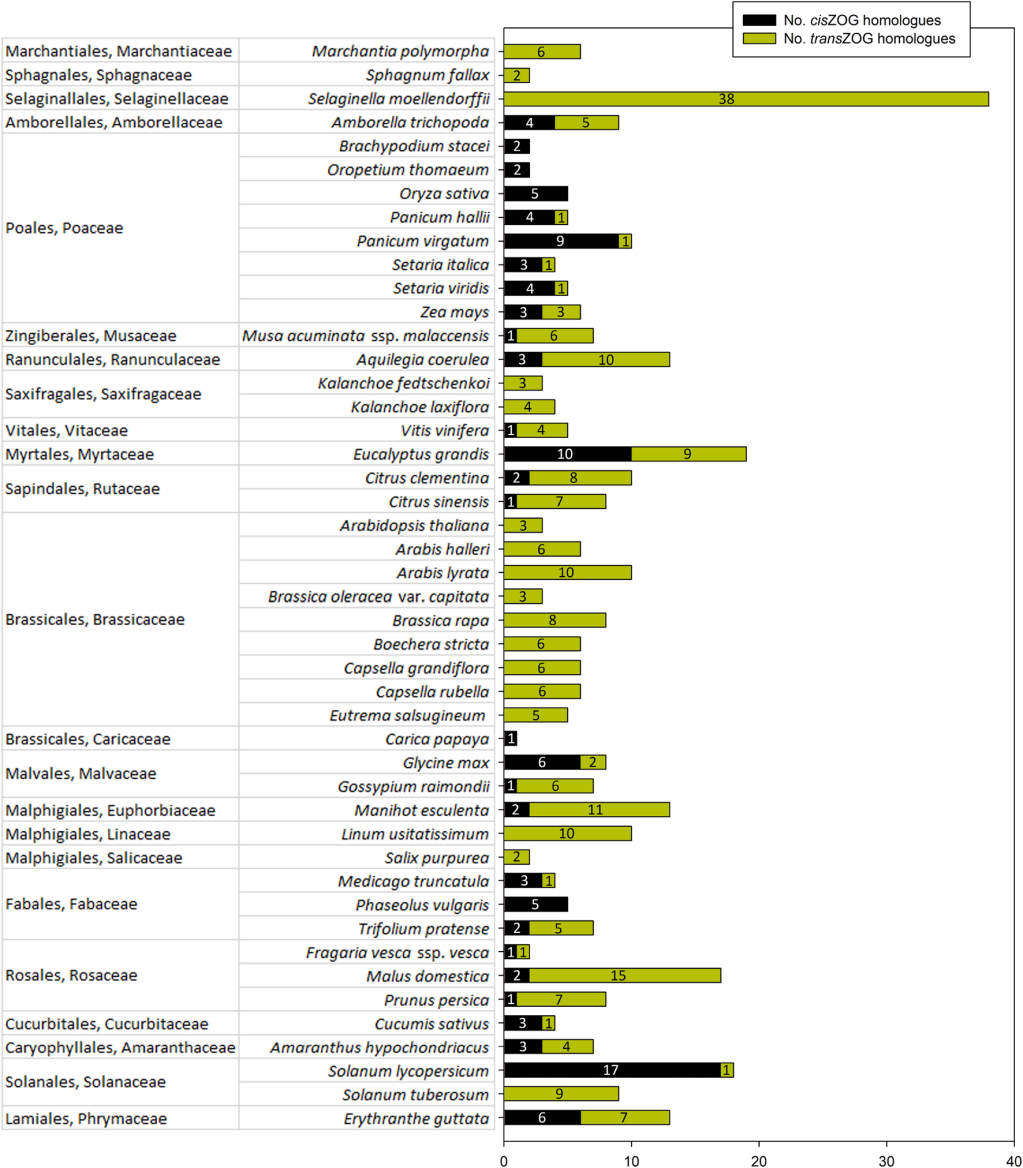
Quantity of *cis*- and *trans*ZOG homologs of angiosperm species from complete genomes (Phytozome v. 12) sorted by phylogenetic system. Species from bryophytes (*Marchantia polymorpha, Sphagnum phallax*) and lycophytes (*Selaginalla moellendorffii*) are shown for comparison with angiosperms.

Most often, one to five *cis*ZOG genes per plant species were revealed. However, in grasses, *Panicum virgatum* contained the most *cis*ZOG homologs in monocots: nine. Four or five homologs were found in other representatives from Poales (*Oryza sativa* and *Panicum hallii*). The highest number represents plants from eudicots, *Solanum lycopersicum* (Solanales) with 17 *cis*ZOG homologs and *Eucalyptus grandis* (Myrtales) with 10 *cis*ZOG homologs (Fig. 2).

**Figure 3:**
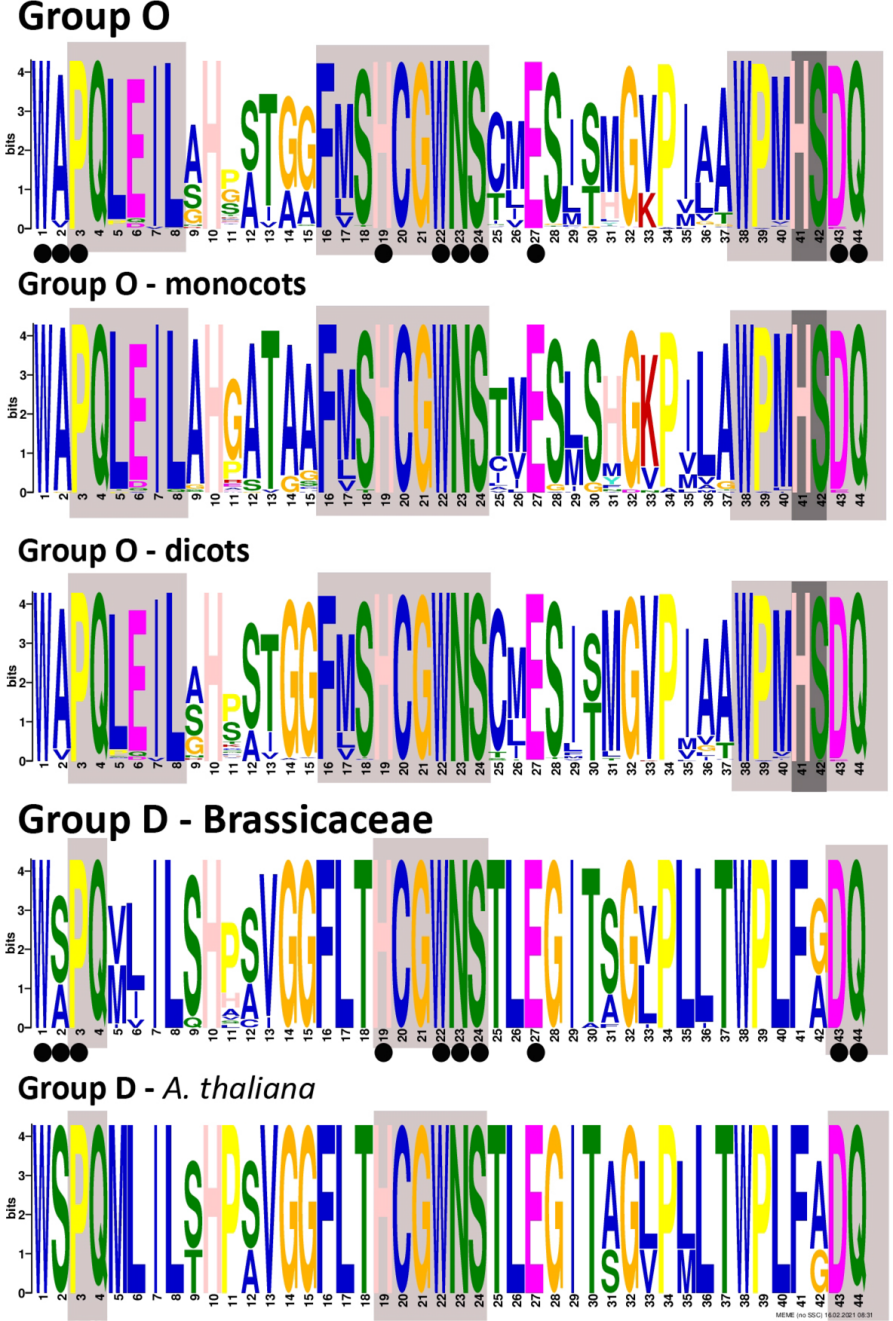
The conserved plant secondary product glycosyltransferase (PSPG) motif in group O in all taxa analyzed, monocots and eudicots in comparison with group D represented by Brassicaceae family. Highly conserved amino acids are shown in boxes (Group O: 1: 5′-PQLEIL-3′, 2: 5′-FMSHCGWNS-3′ and 3: 5′-WPMHSDQ-3′; group D: 1: 5′-PQ-3′, 2: 5′-HCGWNS-3′ and 3: 5′-DQ-3′). Group O—*cis*ZOG is characterized by 5′-HS-3′ in the position 41 and 42 of PSPG. Circles indicate the residues interacting directly with the UDP-sugar based on available crystal structures according to Ref.^9^.

We screened representatives from each of the major evolutionary groups for their main motifs and domains. The *cis*ZOG gene belonging to the O group of glycosyltransferases is characterized by a conserved protein PSPG motif (Fig. 3). This motif is specific for monocots and eudicots and has three well-characterized parts common to both groups (1: 5′-PQLEIL-3′, 2: 5′-FMSHCGWNS-3′ and 3: 5′-WPMHSDQ-3′). A detailed summary of clade/order-specific PSPG motifs is shown in Supplementary Fig. S1 (Amborellales; monocots: Asparagales-Arecales and Poales; dicots: Proteales-Ranunculales-Vitales, Fabales, Rosales, Fagales-Cucurbitales, Malphigiales, Myrtales-Sapindales-Malvales, Brassicales-Caryophyllales-Gentianales, Solanales, Lamiales-Asterales-Apiales). The conserved part were the sugar donor residues, e.g., W in the position 22, D—43 and Q—44 that are positioned to form hydrogen bonds to the sugar part of the donor as described^33^. Lastly mentioned, glutamine (Q) conserved in group D as well, is important for the maximal catalytic efficiency of glucosyl transfer activity^34,35^ and is highly conserved within all UGT groups (A-P as described by Ref.^9^). Furthermore, within group O the sugar donor residues W/P/H/E in the position 1/3/19/27 are invariable across all studied phylogenetically diverse species.

*Trans*ZOG, belonging to the group D have similar PSPG motif (1: 5′-PQ-3′, 2: 5′-HCGWNS-3′ and 3: 5′-DQ-3′), but differ in position 41 and 42, where they have instead of 5′-HS-3′ a 5′-FG-3′ or 5′-FA-3′ (Fig. 3).

Group H (UGT76) known to catalyze N7 and N9 glycosylation of cytokinins differ from group O (*cis*ZOG) in position 5, 7, 11–13, 18, 20, 25, 26, 30, 31, 35–38, 40–42 of PSPG motif. Sugar donor residues are stable.

We identified 15 different conserved motifs shared among related proteins in the whole proteins (Supplementary Fig. S1), and most of the *cis*ZOG genes had a similar intron-phasing distribution.

Furthermore, we searched the Pfam Motif Library and the NCBI Domain Architecture Retrieval Tool for unique motifs characteristic of the ZOG protein and found the most common glycosyltransferase family 28 C-terminal domain (Pfam PF04101; Supplementary Table S1) across angiosperms. Cerato-platanin (Pfam PF07249) was found in four families: Fabaceae, Malvaceae, Amaranthaceae and Solanaceae. Family Solanaceae contains one specific motif, the choline kinase N-terminus (Pfam PF04428), and similarly, members of Fabaceae contain bacterial toxin 8 (Pfam PF15545). Interestingly, *Coffea arabica* (Rubiaceae) contains a putative bacterial lipoprotein (DUF799, Pfam PF05643). Moreover, the sequence similarity is higher within *cis*ZOG genes in monocots than in dicots. The *cis*ZOG protein identity varies from 52 to 96.3% in *Zea mays* or 40.1–96% in *Oryza sativa* cv. *Japonica*. In general, *cis*ZOG sequence identity varies from 23% (*Amborella trichopoda* and *Sorghum bicolor*) to 99.2% (*Solanum penelli* and *Solanum lycopersicum*). There were also differences within one genus: *Nicotiana attenuata* ZOG genes showed 49.2–61.6% sequence identity, *N. sylvestris* 51.4–78%, *N. tabacum* 62.2–78.8 and *N. tomentosiformis* 49.2–77.8%.

### Phylogenetic reconstruction

**Figure 4:**
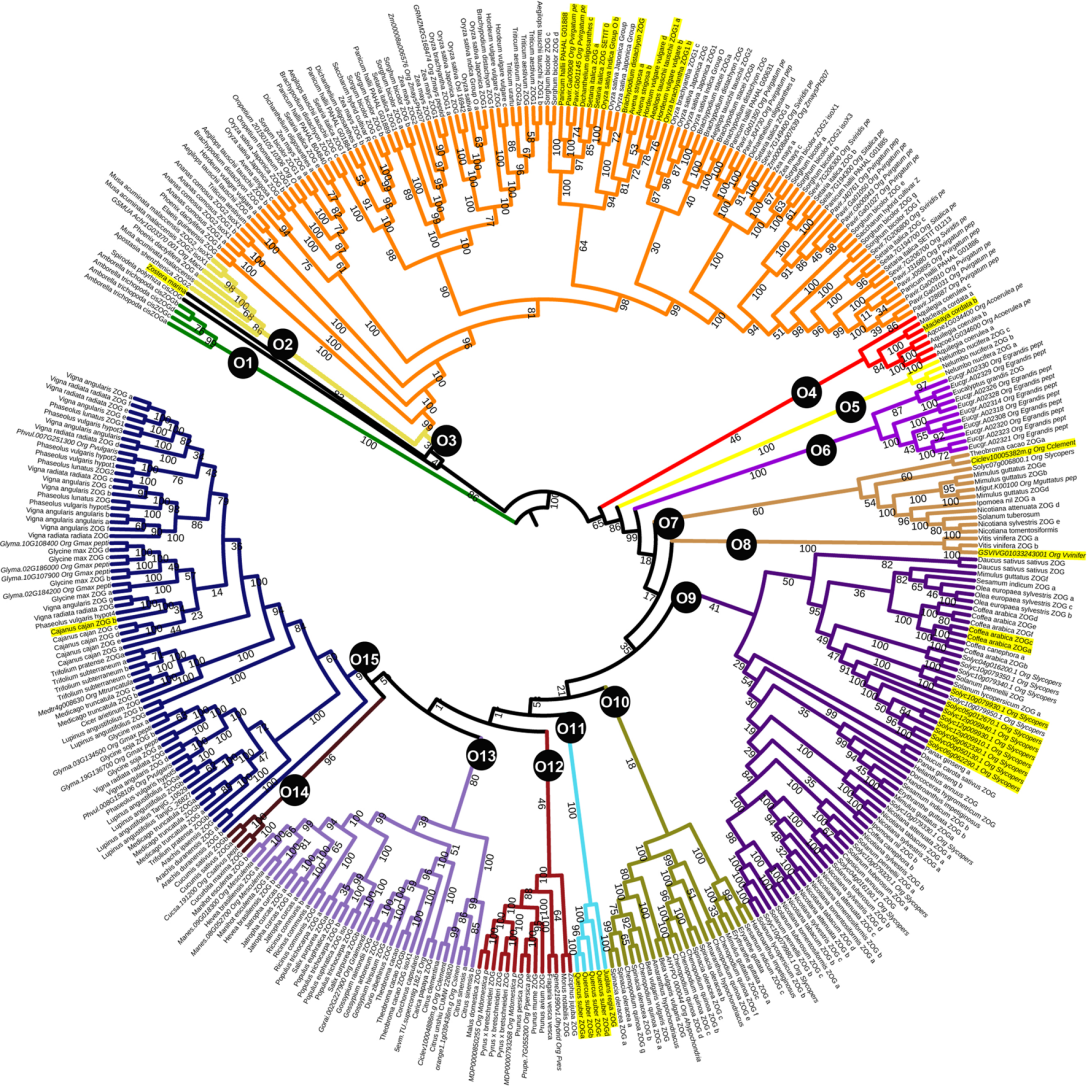
Maximum likelihood phylogenetic tree of *cis*-zeatin *O*-glucosyltransferase based on combined data from Phytozome v12 and GenBank. Twenty-seven species were included in 376 sequences with 1719 positions in the final data set. The ML log likelihood is − 112,690.187027. *Cis*ZOG homologs of *Amborella trichopoda* were used as outgroups. The tree is divided into fourteen main groups: O1. Amborellaceae, O2. Musaceae, O3. Arecaceae-Bromeliaceae-Musaceae-Orchideaceae-Poaceae, O4. Ranunclulaceae, O5. Myrtaceae, O6. Asteraceae-Apiaceae-Araliaceae-Oleaceae-Phrymaceae-Rubiaceae-Solanaceae, O7. Amaranthaceae, O8. Rutaceae-Phrymaceae, O9. Vitaceae, O10. Moraceae-Rhamnaceae-Rosaceae, O11. Cucurbitaceae, O12. Malvaceae-Euphorbiaceae-Rutaceae, O13. Fabaceae, O14. Nelumbonaceae, O15. Fagaceae. Numbers below branches indicate bootstrap support values > 50%. Yellow boxes refer to sequences without a two highly conserved residues, histidine and serine, at positions 41 and 42 in PSPGD domain, italics indicate sequences from Phytozome.

The evolutionary relationships among the *cis*ZOG proteins were determined using maximum likelihood (ML; Fig. 4, simplified Supplementary Fig. S2) and maximum parsimony (MP; Supplementary Fig. S2a,b) analyses based on multiple alignment, producing a phylogenetic tree depicting the relationships among all currently accessible *cis*ZOG sequences. A member of the basal angiosperm *Amborella trichopoda* (Amborellaceae, Amborellales), sister to the rest of the tree in our phylogeny, was used as the outgroup. The first analysis contained 116 sequences of 42 plant species with a total amino acid alignment of 1608 positions in the final data set (not shown). The second analysis with included data from homology searches via NCBI contained 376 sequences for 96 plants and 1719 positions in the aligned matrix (Fig. 4). Three hundred seventy-six sequences were analyzed for the presence of the PSPG motif, and three hundred forty-three contained the motif, i.e., only thirty-three sequences did not contain His and Ser at positions 41 and 42 (Fig. 4). Analyses with full sampling of *cis*ZOG proteins (Fig. 4), selection of fully sequenced genomes only and selection to species with PSPG motif only resulted in the same phylogenetic results. ML and MP analyses revealed 15 distinct groups (O1–O15), MP analysis resulted in more clades (e.g., O4 is divided in 3 clades, O12 to 2 clades), however this finding should to be result of long-branch phenomenon and we can take in mind that these analyses are based on rather artificial selection of available sequence information.

The phylogeny of single *cis*ZOG proteins in an angiosperm lineage is mostly reflected by their taxonomy. Surprisingly, we did not find the *cis*ZOG protein in *Arabidopsis thaliana* or in other representatives of the family Brassicaceae and, in general, in most of the order Brassicales except for *Carica papaya.* However, we also analyzed a data set of fully annotated sequences of 48 species that contained 148 *cis*ZOG sequences (1607 bp), including representatives from Brassicaceae (Supplementary Fig. S3). We revealed that Brassicaceae species clustered together with very short branch lengths and showed different domains in comparison with the rest of the sequences (Supplementary Fig. S3). We performed an additional BLASTP search and found sequence homology with UGT73C. Our BLAST and phylogenetic analyses of annotated *cis*ZOG in *Arabidopsis thaliana* (*cis*ZOG1: AY573820.1, *cis*ZOG2: AY573821 and *cis*ZOG3: AY573822) revealed identity with UDP glucosyltransferase 73C (UGT73C1, UGT73C6 a UGT73C5) belonging to group D but not to group O as demonstrated by other ZOG genes.

The evolutionary history of the *cis*ZOG gene supported independent expansions in monocots and dicots. We recognized two main groups based on clade support values: monocot clade and eudicot clade with 100% (99%) and 62% (65%) BS, respectively. Within monocots (Fig. 1, branch O3), three main basal clades contain Orchideaceae, Musaceae and Arecaceae (BS 83%), Arecaceae (BS 100%) and Bromeliaceae (BS 100%). These clades are followed by seven branches of Poaceae (BS 81–100%). In eudicots, 12 main clades were recognized (Fig. 1, branch O4–O15) with bootstrap support from 80–100%, except for three cases, where BS was lower (branch O10: Amaranthaceae, BS 18%, F: Asteraceae-Apiaceae-Araliaceae-Oleaceae-Phrymaceae-Rubiaceae-Solanaceae, BS 41%, branch O12: Moraceae-Rhamnaceae-Rosaceae, BS46%).

## Discussion

### Phylogenetic diversification of group O UGTs in angiosperms

Glycosyltransferases represent a highly divergent multigene family^6^, where the activities of some subgroups are highly conserved among different plant species, while in others, the substrate specificity shifts with relative ease^5^. After their expansion in vascular plant lineages, UGTs independently acquired their ability to recognize specific compounds as substrates^4^. UGT genes undergo rapid evolution and changes in copy number, making it difficult to identify orthologs and paralogs^36^.

Although CKs have been detected within the plant kingdom ranging from algae to land plants, *cis*ZOG genes have been found only in angiosperms so far (for review see e.g. Refs.^2,37^). In general, ZOG belongs to group O of family 1 UGTs. It contains a low number of proteins (2–9), together with groups B (1–9), F (1–6) and N (1–13), in angiosperms^9^. The *cis*ZOG and *trans*ZOG genes apparently originated and diversified during the evolution of angiosperms, which represents the most recent evolutionary explosion of embryophytes, a lineage that occupied land at least 470 million years ago^38^.

Identification of major protein changes and diversification to two main clades predate eudicot and monocot divergence (Fig. 3, Supplementary Fig. S2). These main clades could have expanded after divergence from their common ancestor. Our phylogeny reconstruction of group O suggests that the *cis*ZOG phylogeny corresponds with a phylogenetic view given by genes reflecting the evolution of angiosperms in general. It is also consistent with^9^ who also showed that the sequences from the different plant species within each phylogenetic group generally have a tendency to cluster together, albeit in some cases they appear to be scattered. On the other hand, the *cis*ZOG homologs do not form a clear separate clade, as described in other protein families^39,40^. This indicates that diversification of the *cis*ZOG gene took place after the diversification of the main angiosperm families, probably within genera or closely related groups.

From one to five *cis*ZOG genes per plant species were revealed. The reconstructed history of *cis*ZOG gene duplication identified by Ref.^24^ presumed that the most common ancestor of grasses contained four copies of the *cis*ZOG gene, and diversification is the result of speciation. During evolution, different numbers of *cis-* and *trans*ZOG genes were apparently duplicated and lost. Duplication of genes is common in plants, where whole genome duplication (WGD) or polyploidy often takes place (polyploid plants range from 30 to 70% in angiosperms). Duplication also played a role in the evolutionary history of angiosperms in determining species richness and diversification. Most of the WGD occurred during periods of environmental instability^41^. These events result in the retention of multiple gene paralogs that may lead to their subfunctionalization, neofunctionalization or redundancy^42^. Although gene duplication is followed by the loss of one of the gene copies, sometimes both copies are retained. In such case, they will initially be redundant, providing an opportunity for one of the paralogs to change function. Existing protein families did not evolve from one common ancestor but under a multiple-birth model^43^. A higher number of paralogs enables avoidance of purifying selection because the activity of some of them is not needed by the cell^44^. Moreover, analyses of WGD within Brassicales showed that gene duplication and loss rates vary across land plants, and different gene families have different probabilities of being retained following a WGD^45^. As shown in *Tarenaya hassleriana* (Brassicales, Cleomaceae), the example of evolutionary fate and functional consequences of a transposition event at the base of Brassicales resulted in the duplication of the floral regulator PISTILLATA^46^. It was previously pointed that gene duplication itself tends to promote divergence of gene expression, most likely just because of their redundancy^44^.

There is a high number of *cis*ZOG isoforms in plants. It corresponds to the results of Ref.^47^, who found conserved alternative splicing events in monocots, particularly across grass species. Isoforms, as the most common result of alternative splicing, are common in plants. Genome-wide transcriptome mapping has revealed the extent of alternative splicing in plants ranging from 42 to 61%^48,49^. Recent data suggest that posttranscriptional regulation, especially alternative splicing, is necessary as a regulatory mechanism for plants to adapt to environmental changes^50^, i.e., similar to paralogs.

### Missing group O UGTs within the family Brassicaceae

The glycosyltransferase superfamily contains 107 UGT genes and 10 UGT pseudogenes in *Arabidopsis thaliana*^5^*.* Our BLAST and phylogenetic analyses of ZOG genes revealed identity with UDP glucosyltransferase 73C (UGT73C1, UGT73C6 and UGT73C5) belonging to group D^6^ in *Arabidopsis thaliana.* The main difference between the *cis*ZOG and UGT73C1 genes is that *cis*ZOG utilizes UDP-glucose as a sugar donor and catalyzes the formation of O-β-D-glucosyl-*cis*-zeatin from *cis*Z^24^, whereas UGT73C1 is primarily involved in the O-glucosylation of *trans*-zeatin and DHZ^26^.

This agreed with the phylogenetic reconstruction of the multigene family 1 UDP glycosyltransferases by Ref.^9^, which revealed two phylogenetic groups, called O and P that are not present in *Arabidopsis thaliana*. To support this finding, the PSPG motif does not contain highly conserved residues^9^*.* The C-terminal region of UGTs contains a 44-amino acid consensus sequence. The PSPG motif. in AY573820.1 is 5′-WSPQMLILTHPAVGGFLTHCGWNSTLEGITSGVPLLTWPL**FG**DQ-3′ (position 41 contains Phe instead of His, and 42 Gly instead of Ser, marked in bold; Fig. 3). The PSPG consensus was originally defined as a signature motif for a plant UGT involved in glycosylation of secondary metabolites^51^. This motif is well conserved and is involved in binding of UDP sugar donors to the enzyme^21,52^. Similar to other UGTs containing the PSPG motif, we found that the *cis*ZOG protein is characterized by only a single or very few introns. This is in contrast to the UGT80 and UGT81 gene families involved in the glycosylation of lipids and sterols and having from 5 to 13 introns^32^. UGTs with the PSPG motif are monophyletic^32^*.* According to Ref.^9^, they were lost at some stage during the plant evolution, which might be due to the massive genome reduction in this species*.* However, we found that the O group is missing not only in *A. thaliana* but also in the whole family Brassicaceae (Figs. 1, 2) as confirmed for: *Arabis halleri, A. lyrata, Brassica oleracea var. capitata, B. rapa, Boechera stricta, Capsella grandiflora, C. rubella* and *Eutrema salsugineum*. Additionally, we did not find it in any member of other 15 families belonging to Brassicales, except for *Carica papaya* from family Caricaceae.

The phylogeny of Brassicales shows three main paleopolyploidization events^53^*.* At-γ is now recognized to be the same duplication as the paleohexapolyploidization detected in both *Carica* and *Vitis*^54,55^. This event is shared by all rosids and potentially all eudicots but is likely not as old as the origin of the angiosperms^53^*.* The *Carica* genome did not contain evidence of having undergone At-β even though both *Arabidopsis* and *Carica* belong to the same order, Brassicales. At-β duplication is a core Brassicales genome duplication. Moreover, *Cleome spinosa* (Brassicales, Cleomaceae) did not find evidence of the At-α event^55^ typical of Brassicaceae. Br-α is typical of the *Brassica* genome^56^.

Within Brassicaceae, the *Carica papaya* genome is unique in other features. An older WGD event in the *Arabidopsis thaliana* lineage (At-β) is not shared by *C. papaya*^56^ but is shared by all sequenced Brassicaceae^57,58^. As summarized by Ref.^59^, the ancestor of all Caricaceae underwent a single WGD event, and chromosome numbers and genome sizes appear stable since^60–62^. Brassicaceae have undergone at least three ancestral polyploidization events^63^; however, no data are available for any Cariaceae genome duplications. Moreover, *Carica papaya* has been recently shown the sole exception not only for the ZOG gene but also for the *Lon* evolutionary history, as the *Lon* gene is the only gene among land plants containing a single copy^64^. Papaya carries the preduplication ancestral *Lon* gene placed at the beginning of the model for *Lon* evolution in plants^64^. We can speculate that *cis*ZOG gene loss in Brassicales occurred before the first WGD (At-β^65^) and diversification of Caricaceae-Moringaceae-Akaniaceae-Tropaeolaceae.

Also representatives of the closely related order Malvales contain the *cis*ZOG gene (*Gossypium raimondii*, *Glycine max*; for details see Fig. 2). Both Brassicales and Malvales are descendants of the paleohexaploid genome common to all eudicots having 21 chromosome pairs resulting from triplication of ancestral *n* = 7^66^. Recently, UGTs belonging to group O were found neither in *Linum ussitatisimum*^67^ nor in lower plant lineages. These results indicate an independent loss of the *cis*ZOG gene in different plant lineages.

### Methylerythritol phosphate and mevalonate biosynthesis pathways

Although zeatin *O*-glucosides have been first discovered in plants already in the last century (*trans*Z *O*-glucoside^68,69^; *cis*Z *O*-glucoside^70,71^), knowledge of when they first appeared during plant evolution is missing. The enzymes involved in *O*-glycosylation of *trans*Z and *cis*Z in plants and their encoding genes have been well characterized in plants^21–24,26^. Whereas only *trans*Z (and partial DHZ) were the substrates of *O-*glucosyltransferase and *O-*xylosyltransferase from lima bean (*Phaseolus lunatus*) and common bean (*Phaseolus vulgaris*), respectively^21,22^, two *O-*glucosyltranferases identified in maize had strict specificity for *cis*Z^23,24^. In maize and other Poaceae representatives, *cis*Z *O*-glucoside and its riboside were reported major CK forms, mostly representing altogether > 80% of the total CKs, in our previous research^17^. Here, we revealed two to nine *cis*ZOG homologs in Poaceae, three found in maize (Fig. 2). Three UGTs capable of *O-*glucosylation are known to occur in *Arabidopsis*, recognizing *trans*Z and DHZ preferentially^26^. It is consistent with the absence of *cis*ZOG gene in the Brassicaceae family reported here and the lack of *cis*Z O-glucosides in selected Brassicaceae species (data not shown).

Moreover, two possible biosynthesis pathways for the isoprenoid moiety of CKs, the MEP and the MVA pathways, occur in plants^11,12^ and play a role in *cis*Z and *trans*Z biosyntheses^10^. The prenyl group of *trans*Z (and iP) is mainly produced through the MEP pathway in plastids^10^. On the other hand, the prenylated adenine moiety of tRNA is typical for *cis*Z, whose formation in *Arabidopsis* may involve the transfer of isoprenoid precursor dimethylallyl diphosphate (DMAPP) from the MVA pathway to tRNA in the cytosol^10^. Distinct origins of DMAPP for *trans*Z and *cis*Z biosynthesis suggest a potentially separate modulation of these CK species levels in plants. The MEP pathway is present in many bacteria and in the chloroplasts of all phototropic organisms. In contrast, the MVA pathway has been found in animals, fungi, plant cytoplasm, archaeobacteria, and some eubacteria^11^. We pointed out that the MEP pathway is phylogenetically old being found in bryophytes (*Marchantia polymorpha* and *Sphagnum phallax*) and lycophytes (*Selaginella moellendorffii*) (Fig. 2). However, the MEP pathway is present in a higher group of plants, angiosperms often have the MVA pathway, and sometimes only the MVA pathway is present (currently found in five species; Fig. 2). Surprisingly, in Brassicaceae, only *trans*Z was found, and the MEP pathway was confirmed (Fig. 3). A similar situation was found in Saxifragales (*Kalanchoe* spp.), only one member of Salicaceae (*Salix purpurea*) and Solanaceae (*Solanum tuberosum*). The pattern in angiosperms is not associated with the evolutionary history of the plants. In general, it is not clear which properties are related to the abundance of the *cis* isomer in a particular plant species^72^. The possibilities include specific environmental conditions, biotic interactions and lifestyle^72^.

## Conclusion

We identified ZOG proteins in over 376 unique accessions in 96 plant species. Our study indicates the expansion of ZOG proteins in both monocots and eudicots. We mainly identified 1–5 putative ZOG proteins in plants with fully sequenced genomes; however, there are exceptions with many more homologs. They were classified into 15 main groups. We confirmed that the *cis*ZOG gene is not present in *Arabidopsis thaliana* and is also missing in the family Brassicaceae and most of the other members of the order Brassicales. Similarly, *cis*ZOG was not found in Malphigiales or Saxifragales. However, except for *Carica papaya* (Brassicales, Caricaceae), only a few representatives from the plant kingdom exist fitting these criteria with results possibly affected by not-so-deeply sequenced genomes. Thus, these data provide a foundation for further detailed studies of the chromosomal locations of ZOG homologs, their secondary structures, expression patterns, as well as interaction partners. Two possible biosynthesis pathways for the isoprenoid moiety of *trans*Z- and *cis*Z-type CKs, the MEP and the MVA ones, occur in plants^11,12^. Based on *cis-* and *trans*ZOG distribution, only the phylogenetically older MEP pathway was found in bryophytes and lycophytes. The MEP pathway occurs in a higher group of plants as well; in angiosperms, it is often involved together with the *cis*ZOG gene in the MVA pathway, and sometimes only the MVA pathway is present. Surprisingly, in Brassicaceae, only *trans*Z was found, and the MEP pathway was confirmed. To date, the pattern in angiosperms is apparently not associated with the evolutionary history of the plants.

## Materials and methods

### Bioinformatic identification of ZOG homologs

We combined two approaches, homology searches via BLASTP from available databases and compilation of all publicly available sequences associated with zeatin O-glucosyltransferase to date. First, the *Zea mays cis*ZOG protein originally reported by Ref.^23^ was used as a query sequence for BLASTP in NCBI protein databases (http://www.ncbi.nlm.nih.gov; not shown separately). Then, we used the homology search tool BLASTP to scan sequences via Phytozome v12^73^ (https://phytozome.jgi.doe.gov) and find more orthologs in different plant species to build the matrix to identify major events of ZOG gene duplication and losses in angiosperms during evolution and to explore evolutionary diversification of ZOGs within the plant kingdom. We performed extensive BLASTP searches using default parameters adjusted to the lowest E-value (< 1e−10) to obtain as many sequences as possible from GenBank. Via these searches, we identified only *cis*ZOG homologs and any representative from Brassicaceae. Second, to collect *trans*ZOG, we searched for ZOG proteins among the annotated genomic sequences from the Phytozome v12 database. Finally, to obtain a more comprehensive set of genes, the Phytozome database was also searched for genes annotated as ‘zeatin O-glucosyltransferase’, and sequences were checked by alignment. The hits obtained from the two searches were then combined, and the redundant sequences were removed. Accession numbers for all retrieved sequences used in analyses are provided in Supplementary Table S2 and alignment in Supplementary Table S3. As a result, we combined sequences representing both attempts into one matrix of *cis*ZOG *O*-glucosyltransferases to unravel the main groups and show patterns of sequence conservation and evolution.

We have two reasons for this exhaustive sampling, rather than only using completely sequenced genomes, although the actual number of species with full genomes available is quite large (Phytozome). First, taxon sampling is thought to impact the accuracy of phylogenetic inference, so we aimed to assess how stable the evolutionary relationships are with the inclusion of additional taxa. Second, we used a complete data set for motif and domain analyses. We eliminated duplicates (i.e., identical sequences of approximately the same length) from all searches. Protein isoforms with the same length were also used because the differential expression patterns producing protein isoforms from various tissues suggested that isoforms could have different biological functions in vivo^74^. However, we also used the IsoCel program^75^ to select from an alternative potential isoform dataset optimized for tree reconstruction. To prevent incorrect inference of gene duplication and losses from species available only from NCBI, we employed for this purpose only sequences obtained from completely sequenced genomes.

### Sequence alignment

Amino acid sequences were aligned using the Clustal Omega algorithm^76^ in the Mobyle platform^77^, with homology detection by HMM–HMM comparisons^78^. We screened data after alignment in the BioEdit program^79^.

Conserved motifs and domains were analyzed in Geneious 11.0.3 (https://www.geneious.com) and MEME 4.11.2 (http://meme-suite.org)^80^. The MEME search was set to identify a maximum of 50 motifs for each protein with a wide sequence motif range from 2 to 50 and a total number of sites ranging from 2 to 600. The Pfam Motif Library^81^ and NCBI Domain Architecture Retrieval Tool^82^ were used to analyze the conserved motifs. The number and arrangement of introns and exons were analyzed using Gene Structure Display Server version 2.0^83^ by aligning the coding sequences with the genomic sequences.

First, we analyzed all sequences independent of their annotations, with no prior assumptions. Later, *cis*ZOG homologs based on sequence identity were checked to determine if they contained the PSPG motif. Because group O contains only two small changes in the PSPG motif and homologs lacking such mutations might still be evolutionarily related to other *cis*ZOGs, we included these sequences in the analyses but marked them to show which do not have PSPG motifs His and Ser at positions 41 and 42. In total, we identified 376 unique accessions in 96 plant species (Supplementary Table S2).

### Phylogenetic and comparative analyses

Maximum likelihood (ML) topology searches were performed in RAxML 8.2.4^84^ to examine differences in optimality between alternative topologies. The analysis involved 376 amino acid sequences and a total of 1711 positions in the final dataset. 1000 replications were run for bootstrap values. To confirm and compare results we used maximum parsimony method (MP). The MP tree was obtained using the Tree-Bisection-Regrafting (TBR) algorithm^85^ with search level 1 in which the initial trees were obtained by the random addition of sequences (10 replicates). Evolutionary analyses were conducted in MEGA7^86^. The bootstrap consensus tree inferred from 500 replicates^87^. Phylogenetic trees were constructed and modified with iTOL v3.4^88^.

## Supplementary Information

Supplementary Figure S1.

Supplementary Figure S2.

Supplementary Figure S3.

Supplementary Table S1.

Supplementary Table S2.

Supplementary Table S3.

Supplementary Table S4.
