# Supplementary material for: Evolutionary diversification of cytokinin-specific glucosyltransferases in angiosperms and enigma of missing cis-zeatin O-glucosyltransferase gene in Brassicaceae
Source: Sci Rep. 2021 Apr 12;11:7885. doi: 10.1038/s41598-021-87047-8 (PMC8041765; doi:10.1038/s41598-021-87047-8)

Evolutionary diversification of cytokinin-specific glucosyltransferases in angiosperms and enigma of missing *cis*-zeatin O-glucosyltransferase gene in Brassicaceae

Lenka Záveská Drábková, David Honys and Václav Motyka

Figure S1. Comparison of the alignment of the plant secondary product glycosyltransferase (PSPG) domain in basal angiosperms, monocots and dicots. Consensus sequences were generated for taxonomic groups (orders).

MONOCOTS

Amborellales

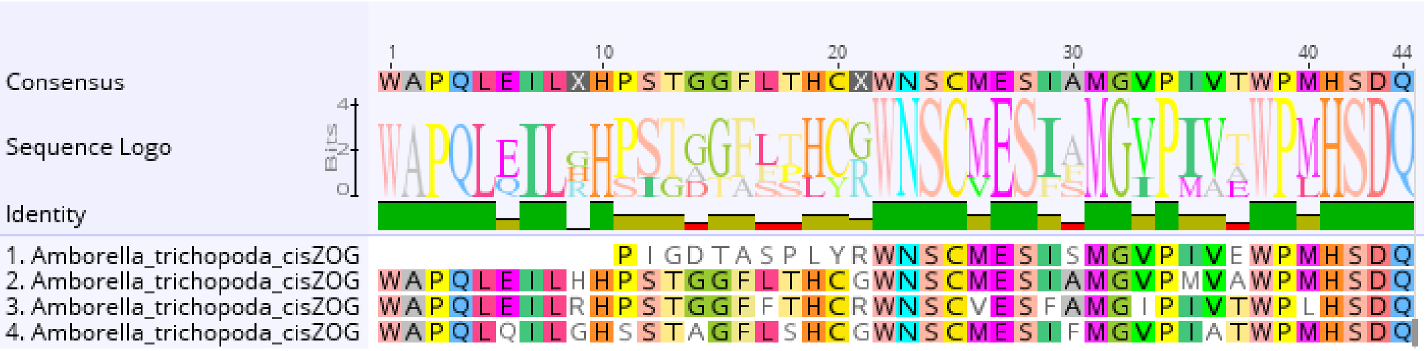

Asparagales, Arecales

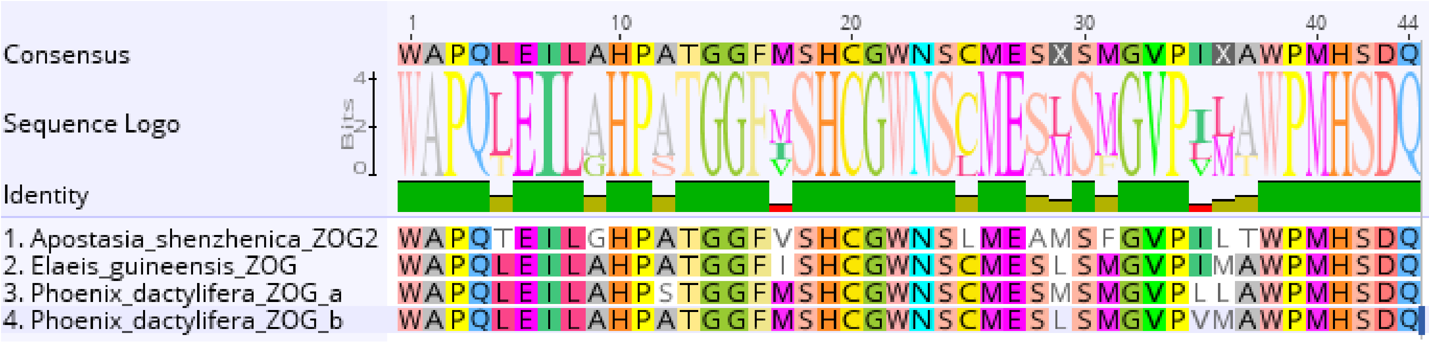

Poales

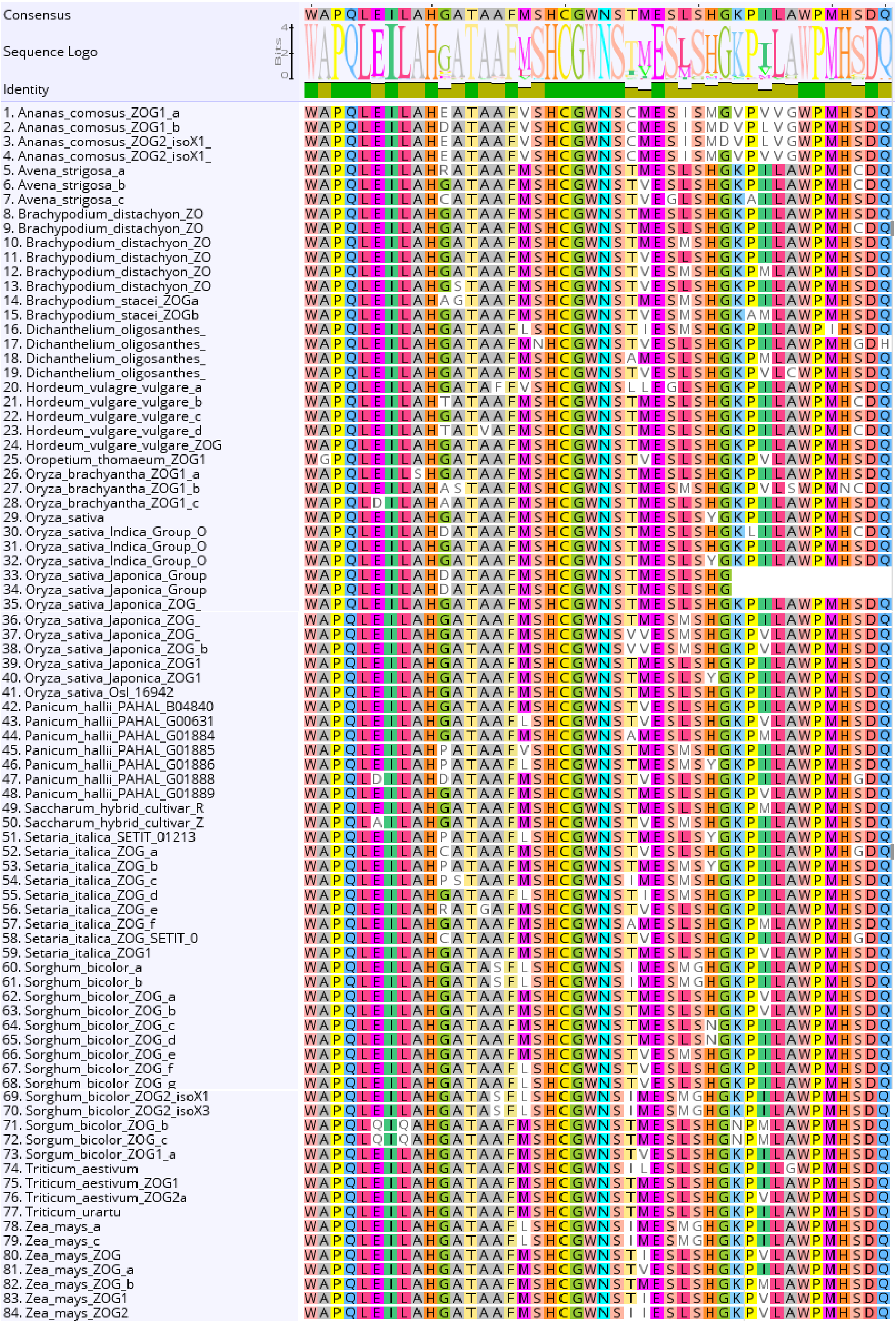

Consensus
WAPQLEILAHGATAAFMSHCGWNSTMESLSHGKPILAWPMHSDQ
Sequence Logo
Bits
Identity
1. Ananas_comosus_ZOG1_a
2. Ananas_comosus_ZOG1_b
3. Ananas_comosus_ZOG2_isoX1_
4. Ananas_comosus_ZOG2_isoX1_
5. Avena_strigosa_a
6. Avena_strigosa_b
7. Avena_strigosa_c
8. Brachypodium_distachyon_ZO
9. Brachypodium_distachyon_ZO
10. Brachypodium_distachyon_ZO
11. Brachypodium_distachyon_ZO
12. Brachypodium_distachyon_ZO
13. Brachypodium_distachyon_ZO
14. Brachypodium_stacei_ZOGa
15. Brachypodium_stacei_ZOGb
16. Dichanthelium_oligosanthes_
17. Dichanthelium_oligosanthes_
18. Dichanthelium_oligosanthes_
19. Dichanthelium_oligosanthes_
20. Hordeum_vulagre_vulgare_a
21. Hordeum_vulgare_vulgare_b
22. Hordeum_vulgare_vulgare_c
23. Hordeum_vulgare_vulgare_d
24. Hordeum_vulgare_vulgare_ZOG
25. Oropetium_thomaeum_ZOG1
26. Oryza_brachyantha_ZOG1_a
27. Oryza_brachyantha_ZOG1_b
28. Oryza_brachyantha_ZOG1_c
29. Oryza_sativa
30. Oryza_sativa_Indica_Group_O
31. Oryza_sativa_Indica_Group_O
32. Oryza_sativa_Indica_Group_O
33. Oryza_sativa_Japonica_Group
34. Oryza_sativa_Japonica_Group
35. Oryza_sativa_Japonica_ZOG_
36. Oryza_sativa_Japonica_ZOG_
37. Oryza_sativa_Japonica_ZOG_
38. Oryza_sativa_Japonica_ZOG_b
39. Oryza_sativa_Japonica_ZOG1
40. Oryza_sativa_Japonica_ZOG1
41. Oryza_sativa_OsI_16942
42. Panicum_hallii_PAHAL_B04840
43. Panicum_hallii_PAHAL_G00631
44. Panicum_hallii_PAHAL_G01884
45. Panicum_hallii_PAHAL_G01885
46. Panicum_hallii_PAHAL_G01886
47. Panicum_hallii_PAHAL_G01888
48. Panicum_hallii_PAHAL_G01889
49. Saccharum_hybrid_cultivar_R
50. Saccharum_hybrid_cultivar_Z
51. Setaria_italica_SETIT_01213
52. Setaria_italica_ZOG_a
53. Setaria_italica_ZOG_b
54. Setaria_italica_ZOG_c
55. Setaria_italica_ZOG_d
56. Setaria_italica_ZOG_e
57. Setaria_italica_ZOG_f
58. Setaria_italica_ZOG_SETIT_0
59. Setaria_italica_ZOG1
60. Sorghum_bicolor_a
61. Sorghum_bicolor_b
62. Sorghum_bicolor_ZOG_a
63. Sorghum_bicolor_ZOG_b
64. Sorghum_bicolor_ZOG_c
65. Sorghum_bicolor_ZOG_d
66. Sorghum_bicolor_ZOG_e
67. Sorghum_bicolor_ZOG_f
68. Sorghum_bicolor_ZOG_g
69. Sorghum_bicolor_ZOG2_isoX1
70. Sorghum_bicolor_ZOG2_isoX3
71. Sorgum_bicolor_ZOG_b
72. Sorgum_bicolor_ZOG_c
73. Sorgum_bicolor_ZOG1_a
74. Triticum_aestivum
75. Triticum_aestivum_ZOG1
76. Triticum_aestivum_ZOG2a
77. Triticum_urartu
78. Zea_mays_a
79. Zea_mays_c
80. Zea_mays_ZOG
81. Zea_mays_ZOG_a
82. Zea_mays_ZOG_b
83. Zea_mays_ZOG1
84. Zea_mays_ZOG2

DICOTS

Proteales, Ranunculales, Vitales

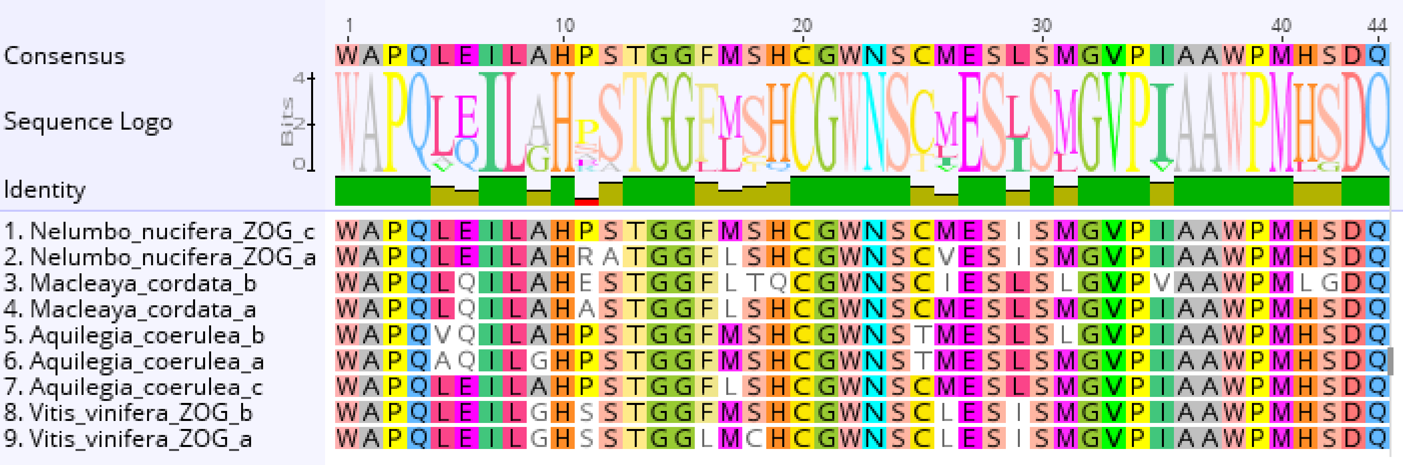

Fabales

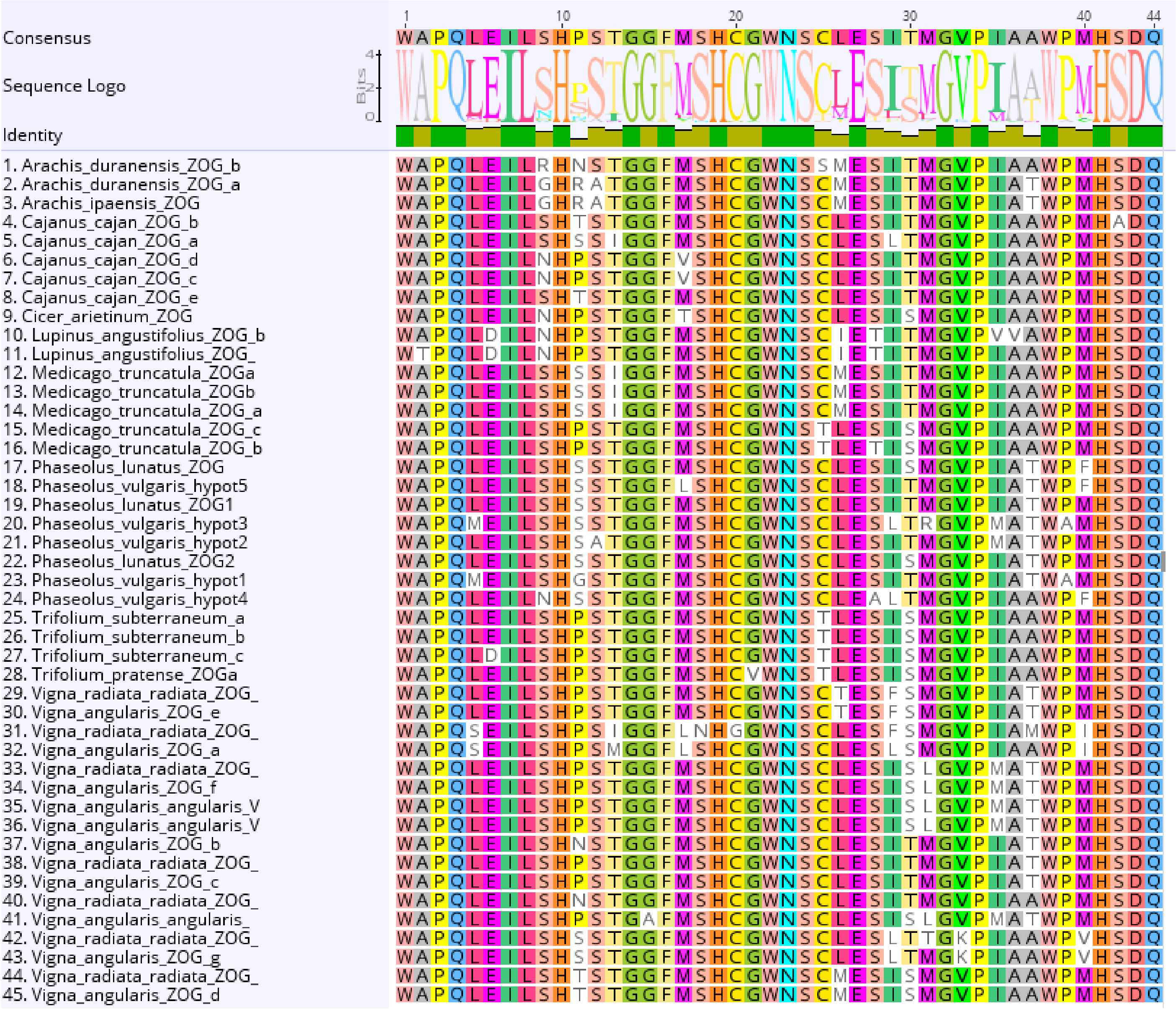

Rosales

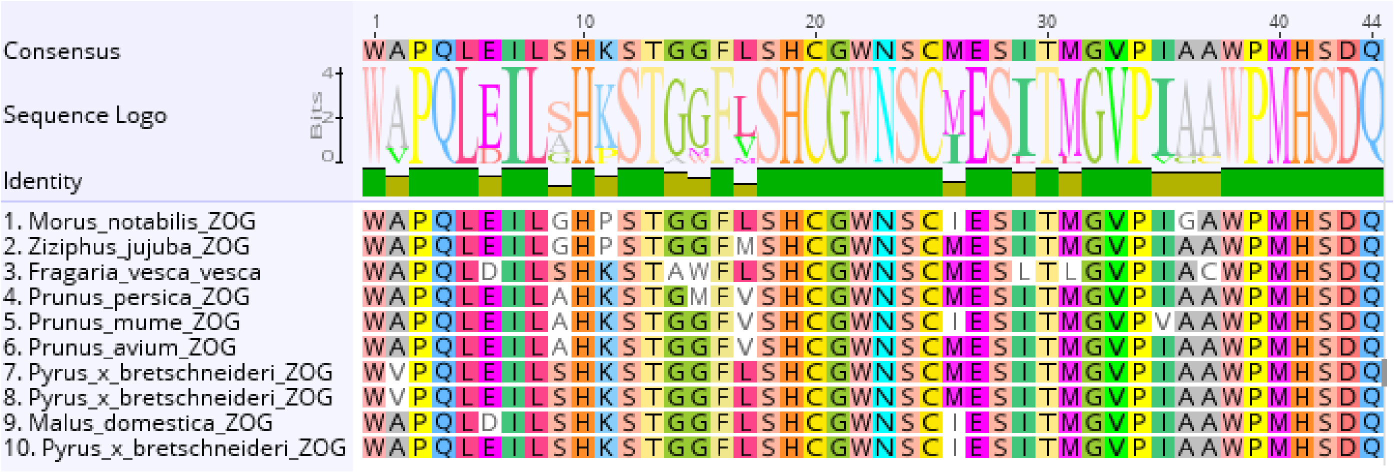

Fagales, Cucurbitales

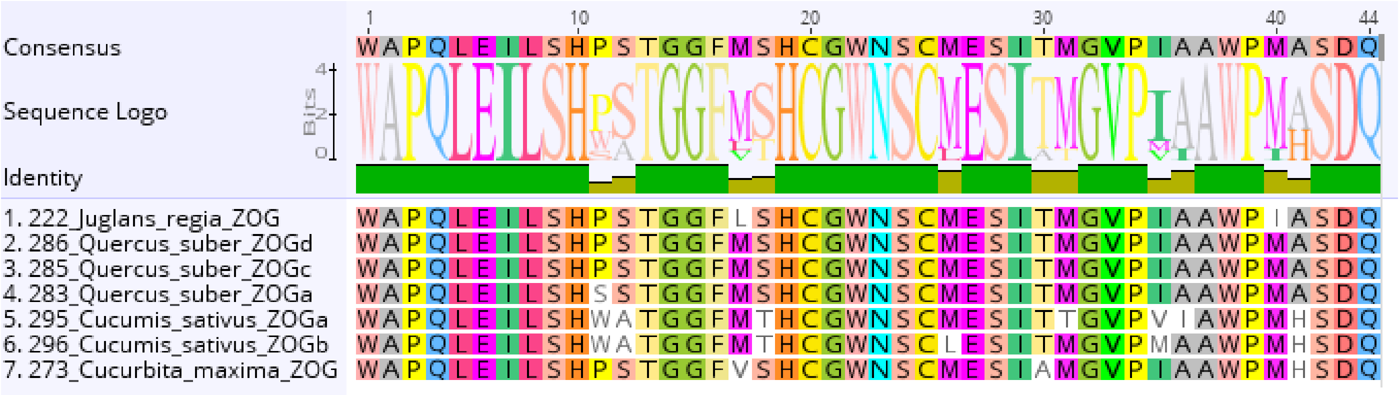

Malphigiales

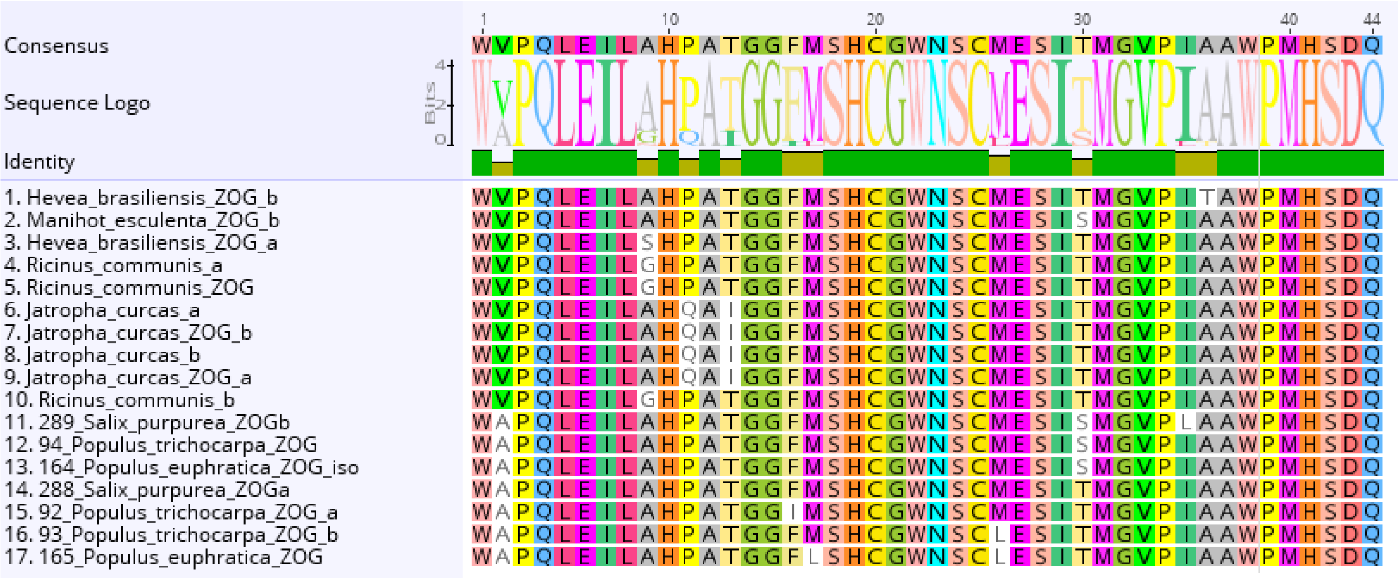

Myrtales, Sapindales, Malvales

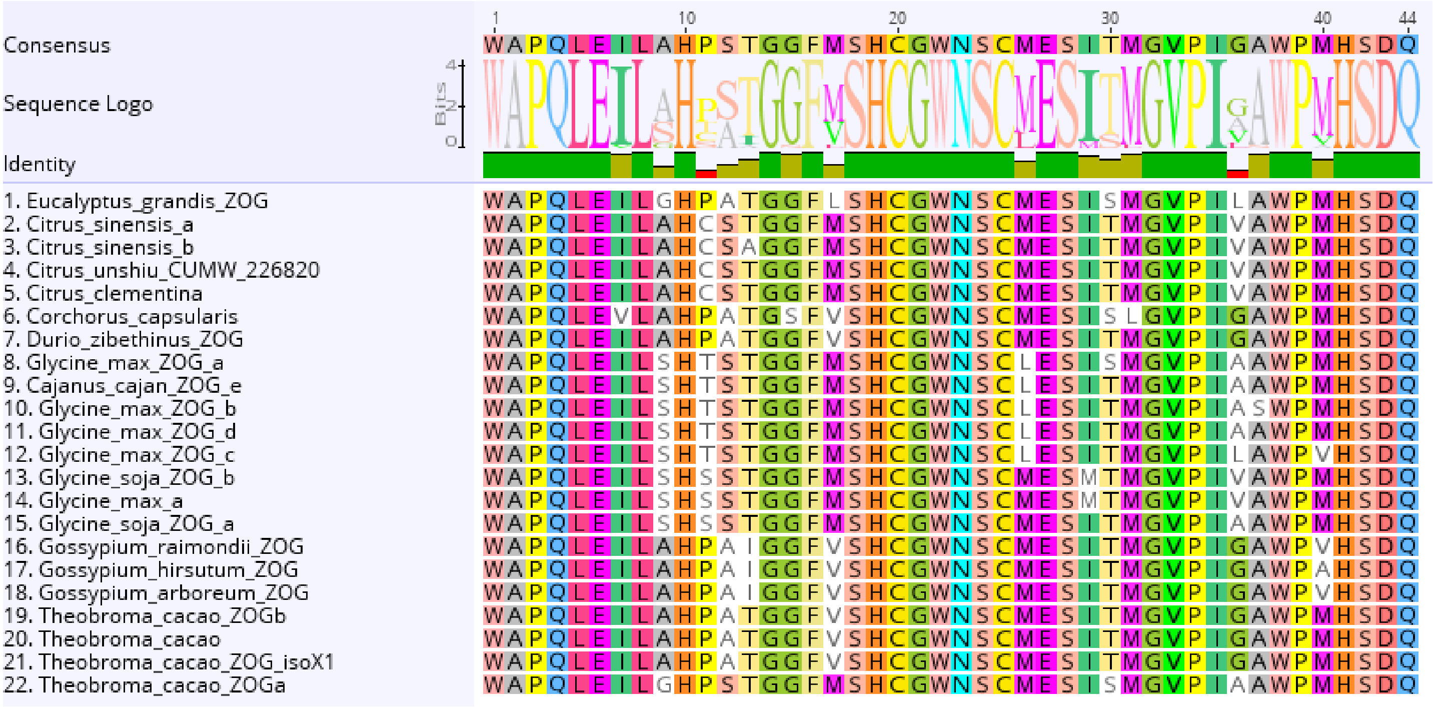

Brassicales, Caryophyllales, Gentianales

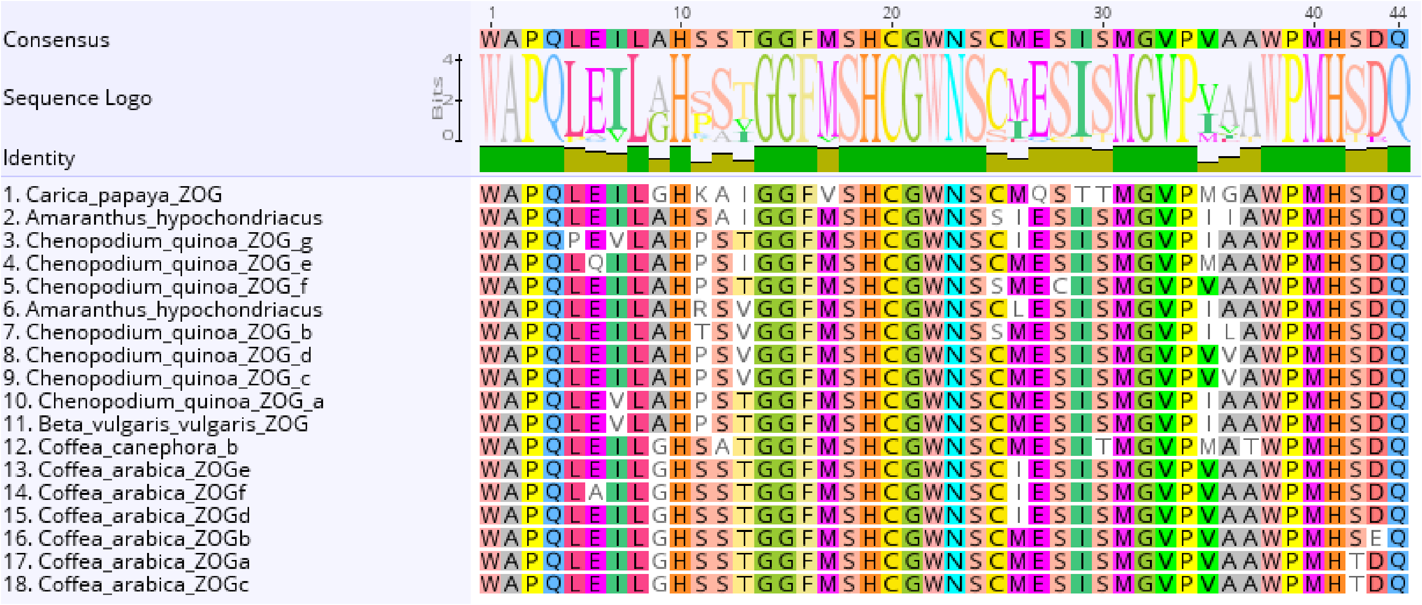

Solanales

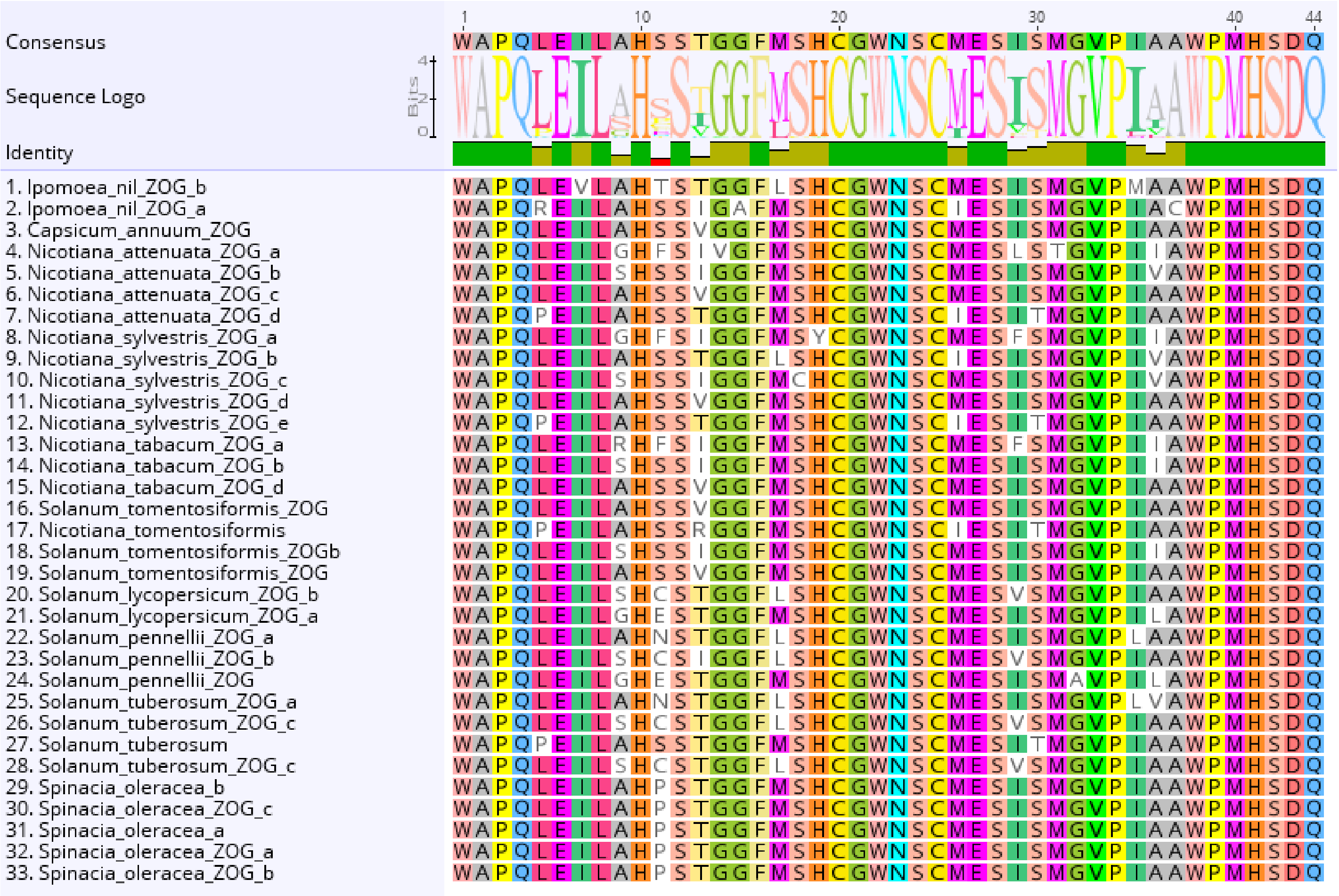

Lamiales, Asterales, Apiales

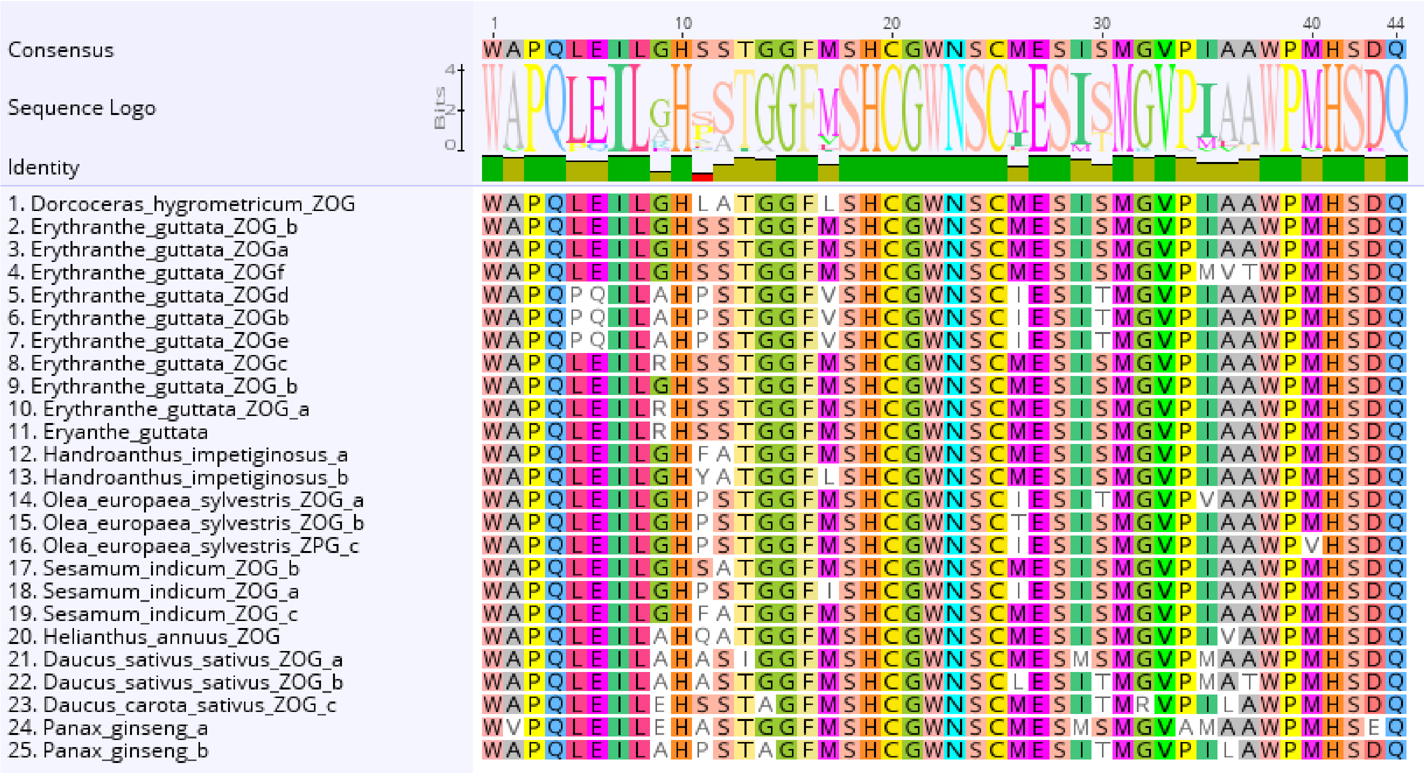

Supplement: Supplementary file 1 — Supplementary Figure S1. [file 41598_2021_87047_MOESM1_ESM.pdf]
