# Supplementary material for: Evolutionary diversification of cytokinin-specific glucosyltransferases in angiosperms and enigma of missing cis-zeatin O-glucosyltransferase gene in Brassicaceae
Source: Sci Rep. 2021 Apr 12;11:7885. doi: 10.1038/s41598-021-87047-8 (PMC8041765; doi:10.1038/s41598-021-87047-8)

Evolutionary diversification of cytokinin-specific glucosyltransferases in angiosperms and enigma of missing *cis*-zeatin O-glucosyltransferase gene in Brassicaceae

Lenka Záveská Drábková, David Honys and Václav Motyka

Figure S2. A. Maximum parsimony phylogenetic tree of *cis*-zeatin O-glucosyltransferase based on combined data from Phytozome v12 and GenBank. Twenty-seven species were included in 376 sequences with 1719 positions in the final data set. Length 21265, CI=0.27, RI=0.75. The *cis*ZOG homologs of *Amborella trichopoda* were used for rooting. Numbers below branches indicate bootstrap support values and fifteen conserved motifs are shown for each sequence. B. Maximum likelihood (left) and maximum parsimony (right) simplified phylogenetic trees. Numbers below branches indicate bootstrap support values >50%. Trees are divided into fifteen main groups corresponding with maximum likelihood tree (Fig. 4): O1. Amborellaceae, O2. Arecaceae-Musaceae-Orchideaceae, O3. Arecaceae-Bromeliaceae-Musaceae-Poaceae, O4. Ranunculaceae, O5. Nelumbonaceae, O6. Myrtaceae, O7. Malvaceae-Phrymaceae-Rutaceae-Solanaceae-Vitaceae, O8. Vitaceae, O9. Apiaceae-Araliaceae-Asteraceae-Convonvulaceae-Phrymaceae-Rubiaceae-Solanaceae, O10. Amaranthaceae-Bignoniaceae-Pedaliaceae-Phrymaceae, O11. Fagaceae-Juglandaceae, O12. Moraceae-Rhamnaceae-Rosaceae, O13. Caricacea-Euphorbiaceae-Malvaceae-Rutaceae-Salicaceae, O14. Cucurbitaceae, O15. Fabaceae. Numbers below branches indicate bootstrap support values and fifteen conserved motifs are shown for each sequence.

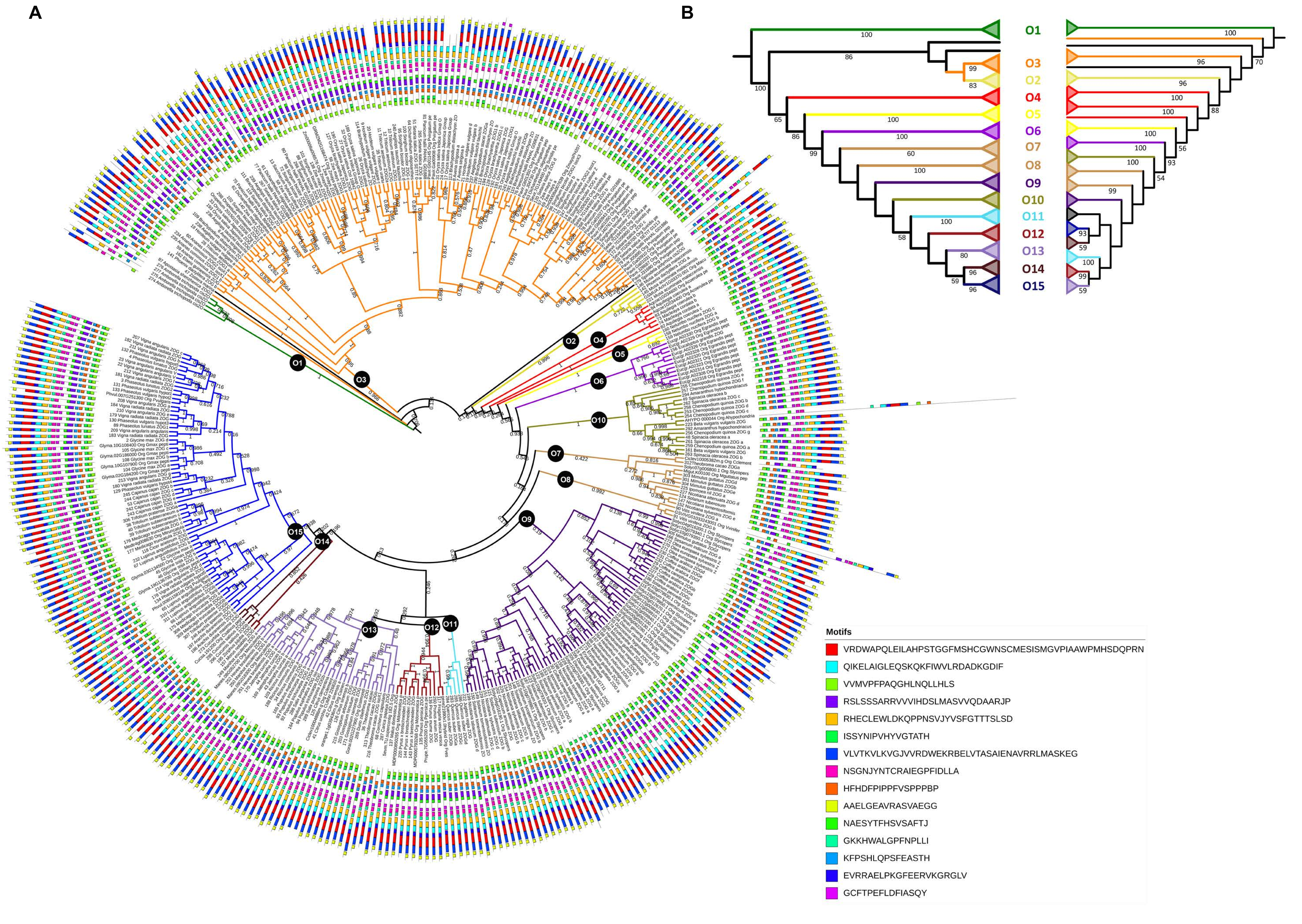

Supplement: Supplementary file 2 — Supplementary Figure S2. [file 41598_2021_87047_MOESM2_ESM.pdf]
