# Supplementary material for: Evolutionary diversification of cytokinin-specific glucosyltransferases in angiosperms and enigma of missing cis-zeatin O-glucosyltransferase gene in Brassicaceae
Source: Sci Rep. 2021 Apr 12;11:7885. doi: 10.1038/s41598-021-87047-8 (PMC8041765; doi:10.1038/s41598-021-87047-8)

Evolutionary diversification of cytokinin-specific glucosyltransferases in angiosperms and enigma of missing *cis*-zeatin O-glucosyltransferase gene in Brassicaceae

Lenka Záveská Drábková, David Honys and Václav Motyka

Figure S3. Maximum likelihood phylogenetic tree of *cis*ZOG and *trans*ZOG (UGT73C for Brassicaceae) based on combined data from Phytozome v12. Numbers below branches indicate bootstrap support values and fifteen conserved motifs are shown for each sequence.

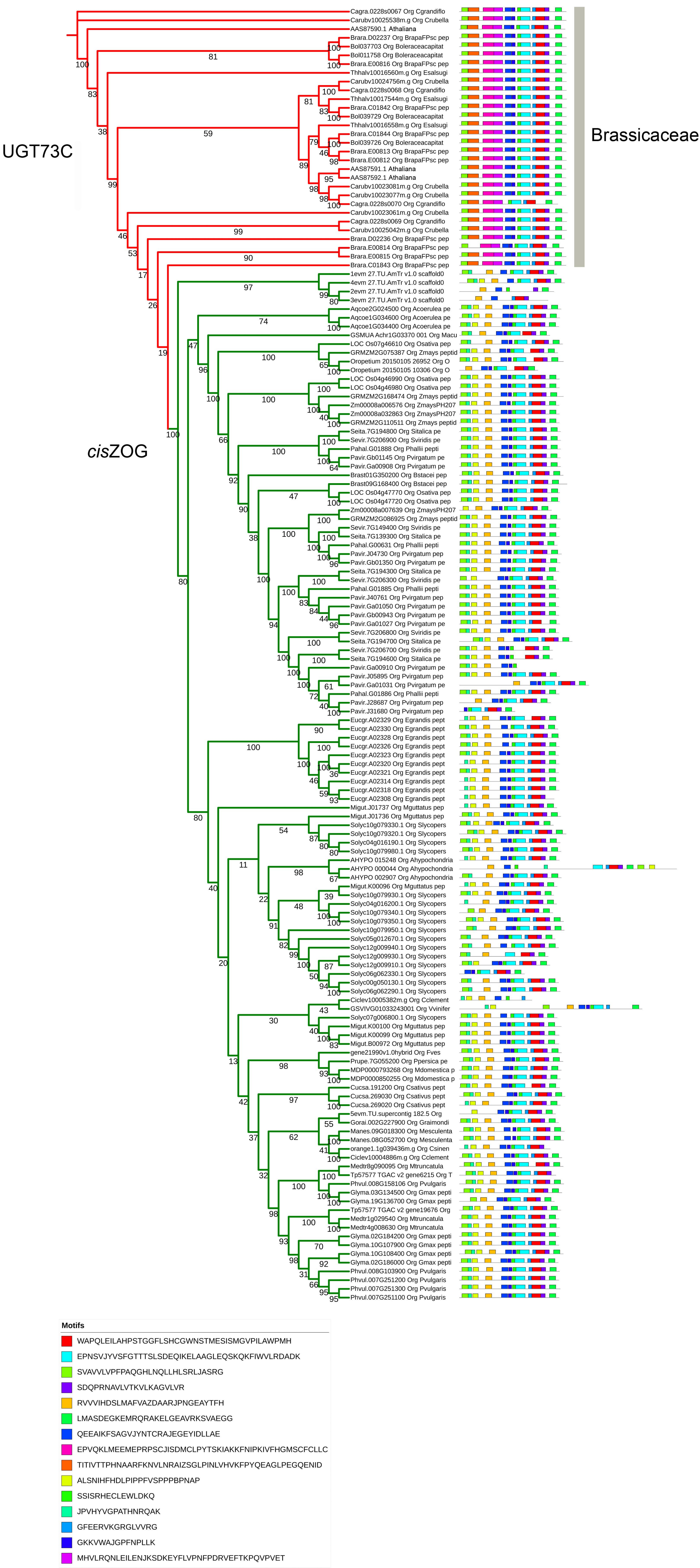

Supplement: Supplementary file 3 — Supplementary Figure S3. [file 41598_2021_87047_MOESM3_ESM.pdf]
